# Vitamin D machinery and metabolism in porcine adipose-derived mesenchymal stem cells

**DOI:** 10.1186/s13287-016-0382-4

**Published:** 2016-08-17

**Authors:** Yovani Llamas Valle, Sami G. Almalki, Devendra K. Agrawal

**Affiliations:** Department of Clinical and Translational Science, Creighton University School of Medicine, Omaha, NE 68178 USA

**Keywords:** Vitamin D_3_, Calcitriol, Adipose-derived mesenchymal stem cell, CYP24A, CYP27B1, Vitamin D receptor

## Abstract

**Background:**

Vitamin D, a hormone once thought to have a role limited to calcium homeostasis and bone mineralization, has pleiotropic effects on different types of cells. Vitamin D receptors are reported in vascular smooth muscle cells, endothelial cells, and cardiomyocytes. Adipose-derived MSCs (ADMSCs) are multipotent cells with the capacity to differentiate into cells of different lineages. To our knowledge, the presence of vitamin D machinery on porcine ADMSCs has not yet been examined. In this study, we investigated the presence of vitamin D machinery and metabolism in ADMSCs by analyzing the expression levels of vitamin D receptor (VDR), vitamin D metabolizing enzymes (CYP24A1 and CYP27B1) after in vitro stimulation with active vitamin D, calcitriol.

**Methods and results:**

ADMSCs isolated from porcine adipose tissue were characterized by positive staining for ADMSC markers, CD44, CD73, and CD90, and negative staining for macrophage marker CD11b and hematopoietic stem cell markers CD34 and CD45, and trilineage differentiation to osteocytes, chondrocytes, and adipocytes. No cytotoxicity was observed when MSCs were stimulated with 0.1–10 nM calcitriol. The ADMSCs were analyzed for mRNA and protein expression of CYP24A1, CYP27B1, and VDR by immunostaining, qPCR, and ELISA. A significant increase (*p* <0.01) in the mRNA expression of CYP24A1, CYP27B1, and VDR was observed after stimulation of ADMSCs with calcitriol (10 nM). The in vitro time-dependent effect of calcitriol (10 nM) on the components of vitamin D machinery in cultured MSCs was determined by qPCR. The VDR and CYP27B1 expression peaked at 3 h and CYP24A1 at 24 h, respectively. The in vitro biosynthesis of 1, 25(OH)_2_D_3_ by ADMSCs was analyzed by ELISA and Western blot. The levels of the active form of vitamin D were significantly decreased once the CYP enzymes were inhibited (*p* <0.01), demonstrating the ability of ADMSCs to convert inactive vitamin D into active vitamin D for cellular action.

**Conclusions:**

Porcine ADMSCs possess vitamin D hydrolases and VDR to metabolize and respond to vitamin D. Hence, in vivo circulating 25-hydroxy vitamin D levels may have a significant role in regulating the differentiation of ADMSCs into different lineages, which might assist in stem cell-based therapy.

## Background

The clinical and biological importance of the steroid hormone, vitamin D, has been extensively studied and linked with multiple classical functions, notably in the skeletal system, where it is responsible for calcium absorption that leads to bone formation [1, 2]. Vitamin D has been characterized as a fat-soluble steroid molecule with one ring open that regulates various downstream signaling pathways to control the transcription of many target genes [3–5]. The deficiency in vitamin D has recently been reported to be associated with non-skeletal muscles illnesses, such as cancer, autoimmune diseases, and cardiovascular disease [6–8]. Currently, the human body can obtain vitamin D from two major sources: dietary intake or by converting 7-dehydrocholesterol in the skin when exposed to ultraviolet B light [9]. In the body, vitamin D binds to vitamin D-binding protein and is transported to the liver, where cholecalciferol is hydroxylated by the enzyme 25-hydroxylase into calcifediol (25-hydroxyvitamin D_3_; 25(OH)D_3_). The 25(OH)D_3_ is then hydroxylated in the kidney by 1α-hydroxylase (CYP27B1) into the active form 1,25(OH)_2_D_3_ (calcitriol). In order to remove any unused 1, 25(OH)_2_D_3_, the 24-hydroxylase (CYP24A1), catabolizes 1, 25(OH)_2_D_3_ into biologically inactive, water-soluble calcitroic acid and excreted from the body [10, 11].

Recent studies have focused on cellular aspects of vitamin D and have demonstrated expression of vitamin D receptor (VDR) and vitamin D hydroxylases in many other cell types such as vascular smooth muscle cells, endothelial cells, and cardiomyocytes [12–14]. However, little is known about how vitamin D may affect mesenchymal stem cells (MSCs). MSCs are multipotent cells that have the capacity to self-renew and differentiate into different lineages [15, 16]. Currently, most research is focused on obtaining MSCs from bone marrow. However, the MSC population only constitutes 0.001–0.01 % of the cell population in bone marrow, about tenfold less than that of hematopoietic stem cells, which makes their availability low in acute clinical conditions [17]. Considering the low availability of MSCs found in the bone marrow, adipose tissue is a promising alternative, due to its abundance in most individuals and that it can be harvested using a simple liposuction procedure. This procedure is less invasive and causes less discomfort and damage to the site of trauma. Adipose tissue has a significantly higher stem cell density than does bone marrow, about 5 % versus 0.01 % of adipose-derived mesenchymal stem cells (ADMSCs) suggesting that a small amount of adipose tissue can yield sufficient stem cells with proliferation and differentiation potential for autologous cell transplantation, especially in acute clinical settings [18]. Further increasing our knowledge on how vitamin D interacts with ADMSCs can provide us with information of how vitamin D can either alter or maintain the same expression of certain receptors in a cell, which in turn either classifies it as a differentiated cell or allow the ADMSCs to keep the stem-like properties for a longer period. To our knowledge, the presence of vitamin D machinery in porcine adipose-derived MSCs has not yet been examined. Pigs are an ideal model due to their cardiovascular and metabolic systems being highly similar to humans in regard to the anatomy, physiology, pharmacology, and pathophysiology. Our laboratory has reported similar findings in human and porcine models [19, 20]. Furthermore, the investigation of MSCs from pigs is a direct translational option since it has been reported that human and porcine MSCs have comparable characteristics and functionality [21]. In this study, we investigated the presence of vitamin D machinery in adipose-derived MSCs by analyzing the expression levels of vitamin D receptor (VDR) and vitamin D metabolizing enzymes (CYP24A1 and CYP27B1) after in vitro stimulation with active vitamin D, calcitriol.

## Methods

### Isolation and culture of porcine ADMSCs

DMSCs were isolated from porcine adipose tissue as previously reported [22]. The Institutional Animal Care and Use Committee approved the research protocol. Briefly, porcine adipose tissue from the anterior abdominal wall of pigs within the ages of 5 ½–6 ½ was collected and transferred to the laboratory from the slaughterhouse under sterile conditions. The ADMSCs from abdominal fat have higher capacity to differentiate into both adipogenic and osteogenic lineages than those adipose tissues obtained from other areas, as reported in the literature [23]. In addition, the proliferation rate was also greater in the abdominal adipose tissue compared to others [24]. The tissue was transported in Dulbecco’s modified Eagle’s medium (DMEM) (Millipore Sigma, St. Louis, MO, USA) with the following antibiotics: 100 mg/mL penicillin (Millipore Sigma, St. Louis, MO, USA) and 100 mg/mL streptomycin (Millipore Sigma, St. Louis, MO, USA). About 10 grams of pig adipose tissue was minced into 2–4 mm pieces with sterile scissors and digested with 15 mL 0.2 % type 1 collagenase (Millipore Sigma, St. Louis, MO, USA) for 2 h at 37 °C. Adding serum-containing media then stopped the collagenase activity. The floating cells were separated from the vascular stromal fraction by centrifugation at 400 g for 10 min. The pellet (stromal vascular fraction) was then filtered through a 100-μm nylon mesh to remove any undigested tissue. The cells were then centrifuged in a 1.077 g/mL Histopaque (Millipore Sigma, St. Louis, MO, USA) density gradient at 400 g for 30 min. The enriched cells were collected from the interphase, washed twice with serum-free medium and then resuspended in complete DMEM containing 10 % fetal bovine serum (FBS) (Gibco, Thermo Fisher Scientific, Waltham, MA, USA), 100 mg/mL penicillin (Millipore Sigma, St. Louis, MO, USA) and 100 mg/mL streptomycin (Millipore Sigma, St. Louis, MO, USA). The cells were then cultured in a 25-cm^2^ flask at 37 °C with 5 % CO_2_/95 % air and 90 % relative humidity. The medium was changed every 2 days. Non-adherent hematopoietic cells were removed by medium change every 24 h for 3 days. Afterward, the culture medium was changed three times per week. Once adherent ADMSCs became confluent, they were then trypsinized using 0.25 % Trypsin–EDTA (Millipore Sigma, St. Louis, MO, USA), and transferred to fresh 25-cm^2^ culture flasks. All experiments were performed using MSCs at 3–6 passages.

### Characterization of ADMSCs

Flow cytometric analysis was performed for the identification of macrophage marker (CD11b), hematopoietic stem cell markers (CD34 and CD45), and ADMSC markers (CD44 and CD90). Cells (1 × 10^6^/mL) were washed with phosphate-buffered saline (PBS) that contained 4 % FBS and were then incubated with the monoclonal antibodies, antihuman CD11b-APC (Clone: ICRF44; eBiosciences, San Diego, CA, USA), antihuman CD34-FITC (Clone: 581; BD Pharmingen, Franklin Lakes, NJ, USA), antihuman CD45-FITC (Clone: 2D1; eBiosciences, San Diego, CA, USA), antihuman CD44-FITC (Clone: IM7; eBiosciences, San Diego, CA, USA) and antihuman CD90-PE (Clone: 5E10; eBiosciences, San Diego, CA, USA) for 30 min at 4 °C in the dark. The dilution and concentration of antibodies were used as specified by the manufacturers. The cells were further washed three times with PBS and resuspended in 500 μl PBS and flow cytometry was performed on a FACS Aria Flow Cytometry System (BD Biosciences, San Jose, CA, USA). FITC, APC and PE-labeled IgGs (BD Pharmingen, Franklin Lakes, NJ, USA) were used as the isotype control as well as positive beads and negative beads [25].

To further observe the presence of surface markers, immunofluorescence staining for CD11b, CD34, CD45, CD73, CD90, and CD105 was performed and observed by fluorescent microscopy. ADMSCs in passage four were seeded in four chamber slides and allowed to reach 60 % confluency. Cells were then fixed with 3.7 % formaldehyde in PBS for 10 min. The fixed monolayer was then rinsed with PBS three times and permeabilized by incubating with 0.1 % Triton-X 100 in PBS for 10 min. After PBS wash, the cells were blocked in 1 % bovine serum albumin (BSA) in PBS for 1 hour and were further incubated with CD11b, CD34, CD45, CD73, CD90, and CD105 antibody (1:100 dilution) for 1 h at room temperature. The cells were then further washed with PBS and incubated with fluorescent-tagged secondary antibody for 30 min. Following this the cells were washed with PBS and mounted in Vectashield with DAPI (Millipore Sigma, St. Louis, MO, USA) and observed using an upright fluorescent microscope. Negative controls were run in parallel in each analysis.

Trilineage differentiation of ADMSCs shows that ADMSCs are capable of differentiating into respective lineages. The ADMSCs were stimulated using STEMPRO osteogenesis, adipogenesis, and chondrogenesis differentiation media using a kit for osteocyte, chondrocyte and adipocyte differentiation (Gibco, Thermo Fisher Scientific, Waltham, MA, USA). For the three lineages, the cells were cultured on chamber slides and the differentiation medium was added at 80 % confluency. The cells were then analyzed for osteogenesis by staining for calcific deposition in the cells using Alizarin S Red after 14 days; adipogenic differentiation by staining for neutral triglycerides and lipids with Oil O Red after 21 days; and chondrogenesis by staining for the synthesis of proteoglycans for 21 days using Alcian blue. Following staining, cells were observed under a bright field microscope. All the histochemical reagents were obtained from Millipore Sigma (St. Louis, MO, USA).

### Analysis of vitamin D machinery

The cytotoxicity of calcitriol on ADMSCs was determined using a Vybrant Cytotoxicity Assay Kit (Molecular Probes, Eugene, OR, USA). The cells were plated in a 96-well microplate with the density of 500 and 1000 cells/well in a 50 μl volume. Cells were then stimulated with different concentrations of calcitriol (0.1–100nM) obtained from Millipore Sigma (St. Louis, MO, USA) for 24 h and 48 h. The Vybrant Cytotoxicity Assay Kit was used to monitor the release of cytosolic enzyme glucose-6-phosphate dehydrogenase (G6PD) from damaged cells into the surrounding medium. It was then quantitatively determined according to the manufacturer’s instructions by measuring the absorbance at 450 nm.

Total RNA was isolated from treated ADMC with Trizol reagent (Millipore Sigma, St. Louis, MO, USA) after treating the cells with 10 nM of calcitriol for 0, 3, 6, 12, and 24 h (n = 3/group) [26]. The yield of RNA was quantified using a NanoDrop (Thermo Fisher Scientific, Waltham, MA, USA). First-strand cDNA synthesis was performed using 1 μg total RNA with oligo(dT) (1 μg), 5 × reaction buffer, MgCl_2_, dNTP mix, RNAse inhibitor and Improm-II reverse transcriptase as per instructed by the kit (Promega, Madison, WI, USA). Following the first-strand synthesis, real-time PCR analysis was performed for the identification of CYP27B1, CYP24A1, VDR, using 8 μl cDNA, 10 μl SYBR green PCR Master Mix (Bio-Rad Laboratories, Hercules, CA, USA) and forward and reverse primers (10 pmol/μl) (Integrated DNA Technologies, San Diego, CA, USA) using a Real-Time PCR system (CFX96, Bio-Rad Laboratories, Hercules, CA, USA). The specificity of the primers was analyzed by running a melting curve. The PCR cycling conditions were 5 min at 95 °C for initial denaturation, 40 cycles of 30 s at 95 °C, 30 s at 52–58 °C (depending upon the primer annealing temperatures) and 30 sec at 72 °C. Each real-time PCR was carried out using three individual samples in duplicates and the threshold cycle values were averaged. Calculations of relative normalized gene expression were performed using the Bio-Rad CFX manager software based on the ^ΔΔ^Ct method. The results were normalized against housekeeping gene: glyceraldehyde-3-phosphate dehydrogenase (GAPDH). Statistical analysis of real-time quantitative PCR results was performed using a one-way ANOVA test.

Immunofluorescent staining for CYP24A1, CYP27B1, and VDR was performed. The treated cells were fixed with 3.7 % formaldehyde in PBS for 10 min. The fixed monolayer of the cells were rinsed with PBS and permeabilized with 0.1 % Triton-X 100 in PBS for 10 min. After PBS wash, the cells were blocked in 1 % BSA in PBS for 1 h and were further incubated with CYP27B1, CYP24A1, and VDR antibodies (1:100 dilution; Santa Cruz Biotechnology, Dallas, TX, USA) for 1 h at RT. The cells were then further washed with PBS and incubated with fluorescent-tagged secondary antibody for 30 min. Finally, the cells were again washed with PBS then mounted in Vectashield with DAPI (Vector Laboratories, Burlingame, CA, USA) and visualized using an upright fluorescent microscope (Olympus BX51, St. Louis, MO, USA). Negative controls were employed in each analysis by omitting calcitriol as a treatment.

Cytochrome P450 24A1 (CYP24A1), and Cytochrome P450 27B1 (CYP27B1) ELISA kits (MyBioSource, San Diego, CA, USA) were used to quantify CYP24A1 and CYP27B1 levels after stimulation of ADMSCs with calcitriol at 0, 3, 6, 12, and 24 h. ADMSCs were detached using trypsin and then collected by centrifugation. The cells were then washed three times with cold PBS before resuspension in PBS. The cells were then lysed through ultrasonication. Next, they were centrifuged at 1500 g for 10 min at 2–8 °C to remove cellular debris. The cells were then added to 96-well plates and assessed according to the manufacturer’s instructions with the absorbance measured at 450 nm on a plate reader.

MSCs were cultivated in 25-cm^2^ culture flasks until 80 % confluence. Cells were then treated with or without 1 nM calcifediol (25(OH)D_3_), with or without 1 μM CYP inhibitor ketoconazole in serum-free medium. After 24 h treatment, the media was collected from each well. The 1,25(OH)_2_D_3_ levels in the media were quantitatively determined with a 1,25(OH)_2_D_3_ ELISA kit (MyBioSource, San Diego, CA, USA), according to the manufacturer’s instructions.

## Results

### ADMSC characterization

**Fig. 1:**
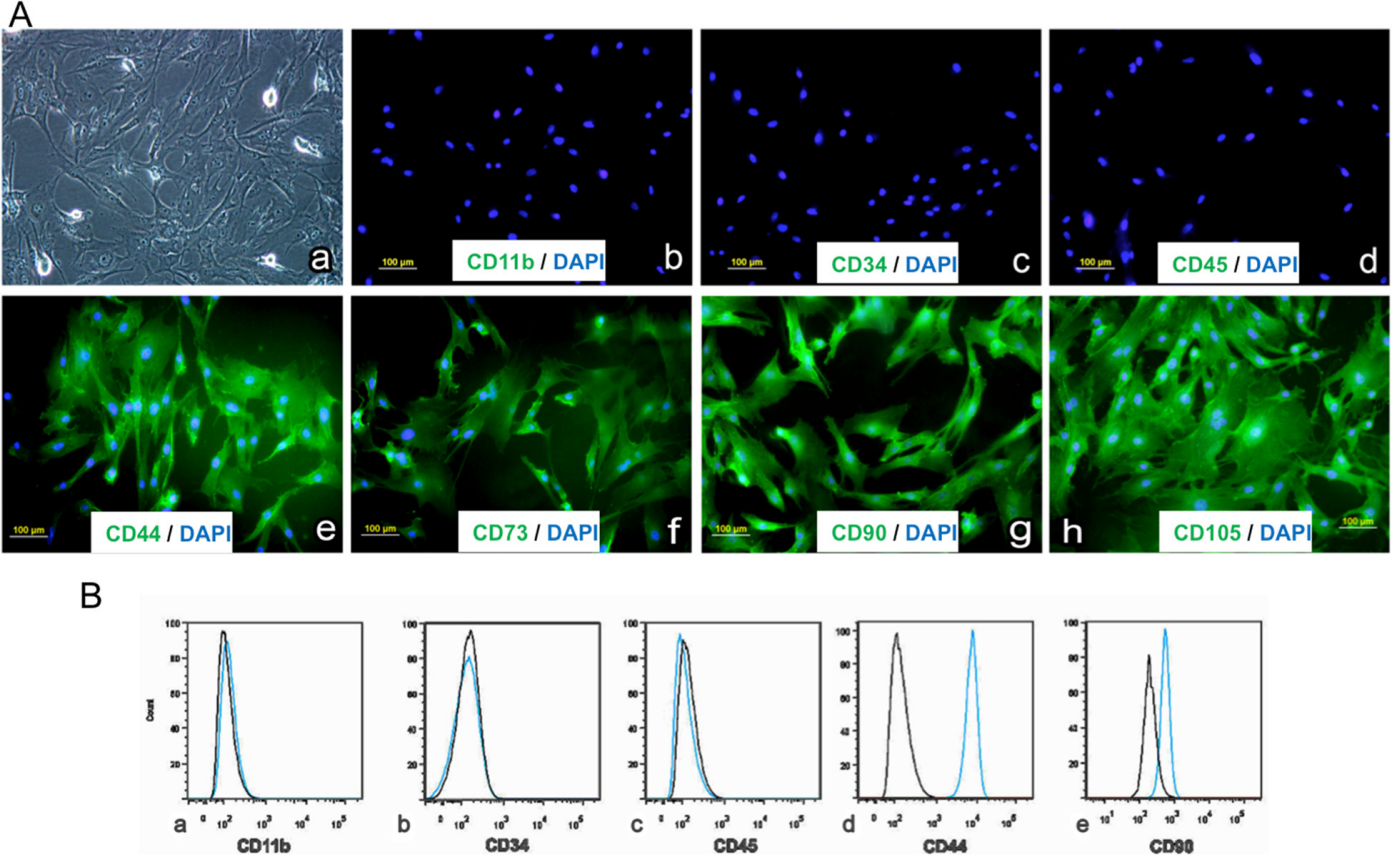
Characterization of ADMSCs using surface markers. **a** Phase contrast image showing morphology of adipose-derived MSCs (**a**), immunostaining data showing negative staining of macrophage marker CD11b (**b**) and hematopoietic stem cell marker CD34 (**c**) and CD45 (**d**); and positive staining for CD44 (**e**), CD73 (**f**), CD90 (**g**), CD105 (**h**), which are the characteristics of ADMSCs. **B** Flow cytometry data showing negative expression of CD11b (**a**), CD34 (**b**), CD45 (**c**) and positive expression of CD44 (**d**), and CD90 (**e**) in the cultured cells. The *black histogram* represents the isotype control and stained cells are represented by the *blue histogram* (*n* = 3)

The plastic adherent cells showed fibroblastoid morphology and stained positively for CD44, CD73 (5′-nucleotidase), CD90 (Thy-1), and CD105 (Endoglin). Contamination with other cells was ruled out by negative reactivity to CD11b (macrophage marker), CD34, and CD45 (hematopoietic stem cell markers) (Fig. 1A). The results were further confirmed with immunophenotyping with flow cytometric analysis. The cells expressed CD44 and CD90, further indicating that the cells originate from a mesenchymal lineage (Figs. 1Bd-e). The frequent change of medium after seeding was effective in preventing the attachment of hematopoietic stem cells and other non-adherent cells, and thus the absence of CD11b+, CD34+ and CD45+ in the population of ADMSCs.

**Fig. 2:**
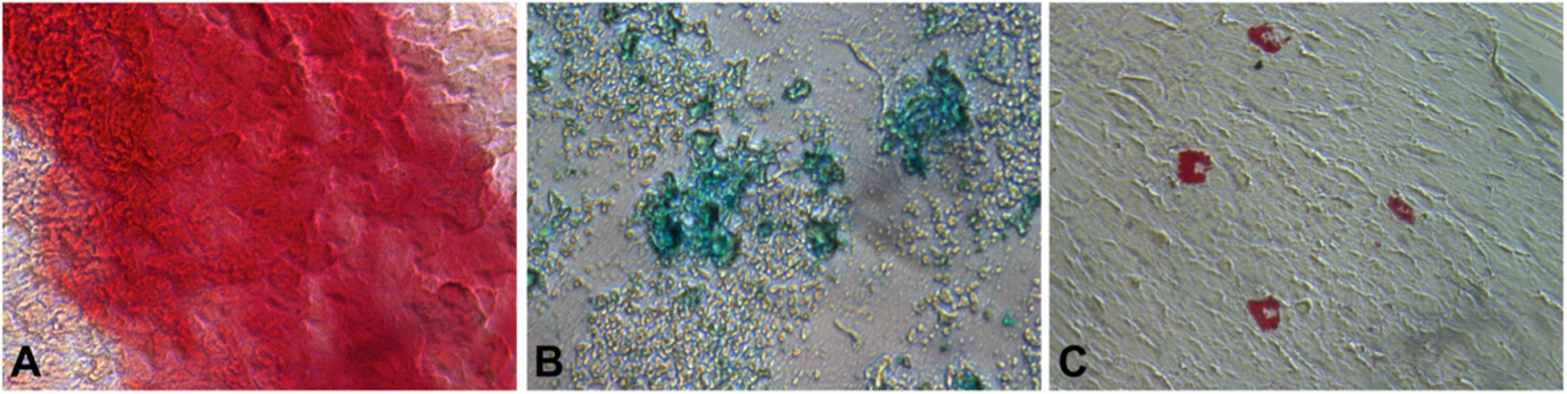
Trilineage-differentiating capability of ADMSCs. The isolated ADMSCs were stimulated with specific differentiation media to examine their trilineage differentiation: (**a**) Alizarin red staining showing the presence of calcium deposition by cells, demonstrating differentiation of ADMSCs to osteogenic lineage, (**b**) Alcian blue staining indicating synthesis of proteoglycans by chondrocytes showing cell differentiation to chondrogenic lineage, and (**c**) Oil Red O staining indicating the synthesis of neutral triglycerides and lipids, showing differentiation of ADMSCs to adipocytes

Cells stained with Alizarin S Red for 14 days showed positive staining for osteogenesis (Fig. 2a). The adipogenic and chondrogenic differentiation of the cells was confirmed by the staining for 21 days with Oil O Red (Fig. 2b) and Alcian blue (Fig. 2c), respectively. The cells were observed under a bright field microscope after staining. The immunophenotyping coupled with trilineage differentiation demonstrated mesenchymal multipotency and effectively confirmed isolated cells to be ADMSCs.

### Analysis of vitamin D machinery

**Fig. 3:**
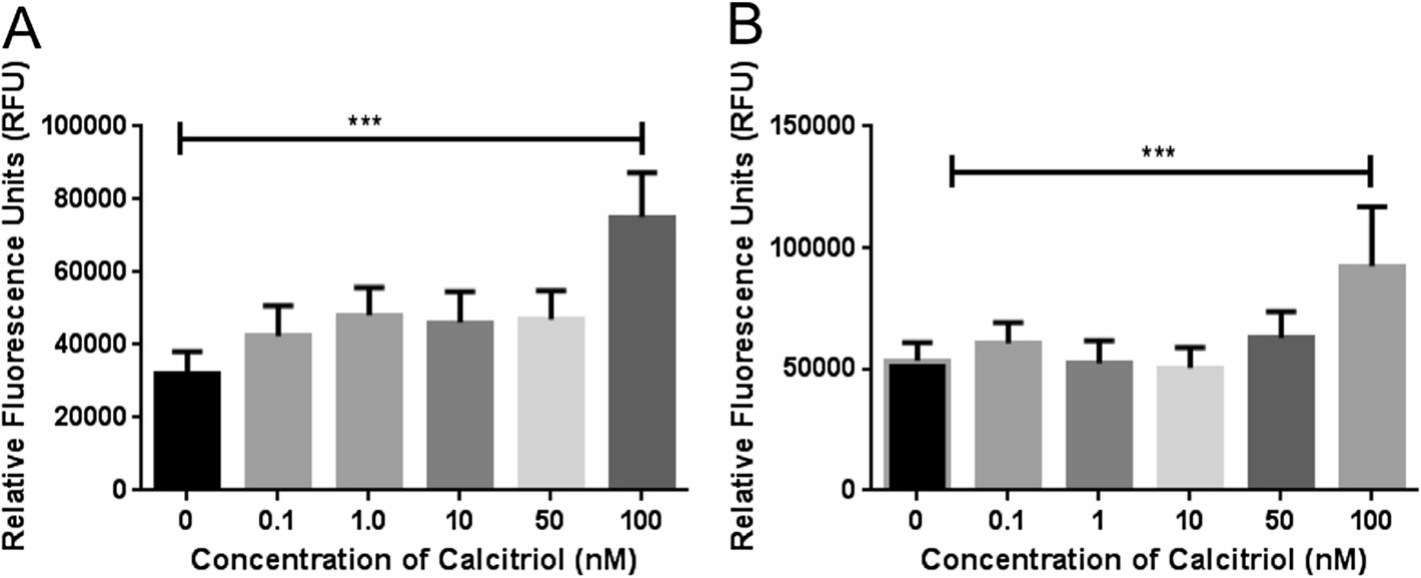
Dose-dependent effect of calcitriol on cytotoxicity. The cytotoxic effect of calcitriol was examined by detecting dead and dying cells using the Vybrant Cytotoxicity Assay Kit after stimulation of ADMSCs with varying concentrations of calcitriol. ADMSCs were treated with 0 nM, 0.1 nM, 1 nM, 10 nM, 50 nM, and 100 nM calcitriol for 24 h (**a**) and 48 h (**b**), respectively and further assayed for glucose 6-phosphate dehydrogenase release. The fluorescence was measured using a microplate reader with excitation/emission at 530/590 nM

The cytotoxicity assay was performed to examine the dose of calcitriol that induce cytotoxicity in ADMSCs. Calcitriol induced a dose-dependent effect when the ADMSCs were stimulated with a concentration that ranged from 0.1–100 nM (Fig. 3) during 24 hours (Fig. 3a) and 48 hours (Fig. 3b). A large amount of cytosolic enzyme, G6PD, was released from the cells stimulated with 100 nM calcitriol (Fig. 3a and b) compared to the cells stimulated with 0–50 nM calcitriol (Fig. 3a and b); there was a difference in the release of G6PD at 0–50 nM calcitriol. Since calcitriol induced cytotoxicity at 100 nM and also the circulating amount of calcitriol in the human body ranges from 0.1 to 10 nM, a dose of 10 nM calcitriol was selected in later experiments.

**Fig. 4:**
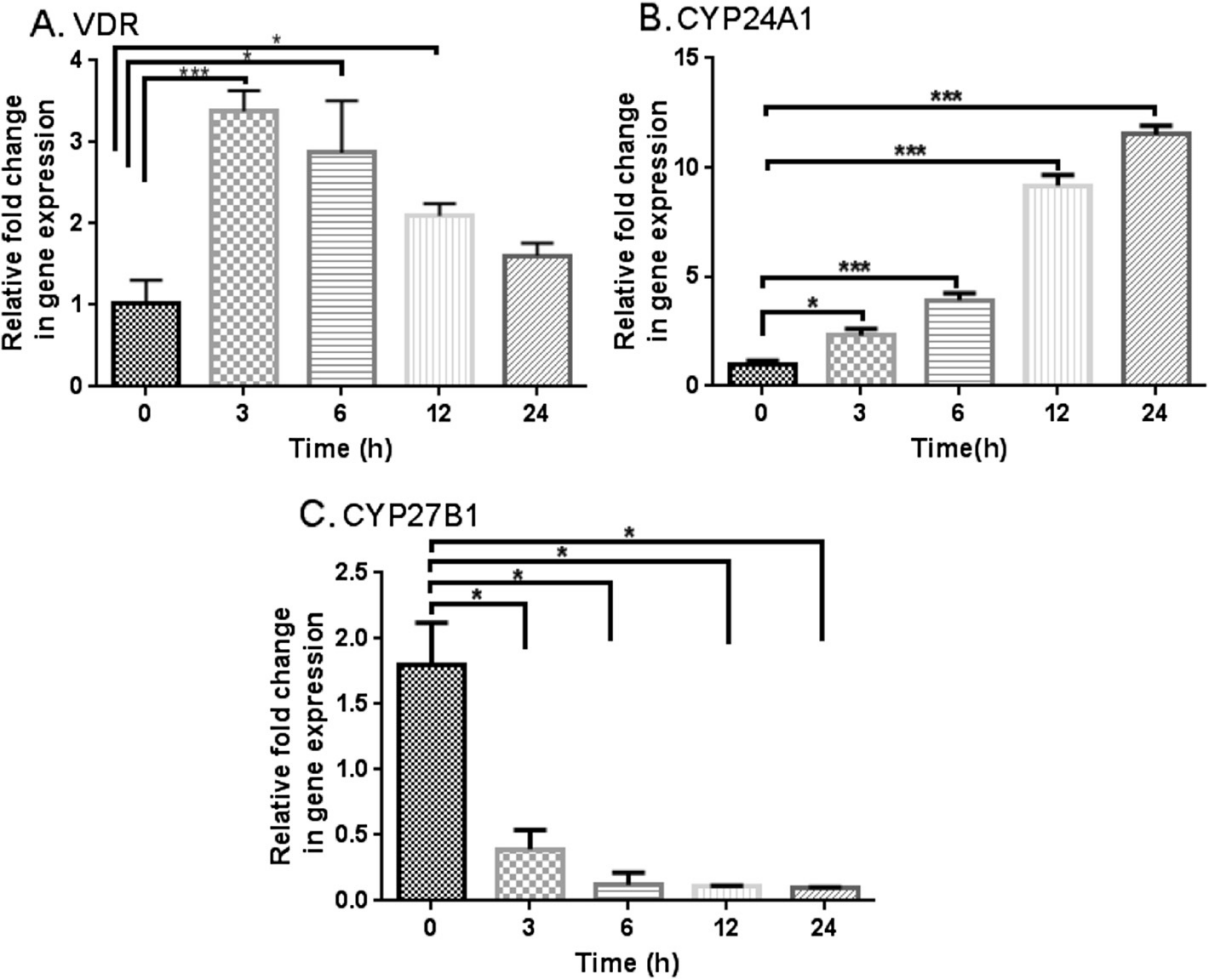
Effect of calcitriol on mRNA transcripts for VDR, CYP24A1 and CYP27B1. Real-time PCR data showing fold change in the expression of VDR (**a**), CYP24A1 (**b**) and CYP27B1 (**c**) after stimulation with calcitriol (10 nM) for 0 h, 3 h, 6 h, 12 h, and 24 h respectively (^***^ *p* = 0.0001, ^**^ *p* < 0.01, ^*^ *p* < 0.05, *n* = 3)

Real-time PCR revealed the expression of the vitamin D machinery genes VDR, CYP24A1, and CYP27B1 (Fig. 4a-c), after stimulation with calcitriol (10 nM) for 0, 3, 6, 12, and 24 h, respectively. There was a significant change in the VDR expression when cells were treated at 3, 6, and 12 h, showing the largest increase at 3–6 h (*p* = 0.0001) followed by a drop at 24 h (Fig. 4a). However, there was time-dependent increase in CYP24A1 with the greatest expression occurring at 24 h (Fig. 4b). In parallel, expression of CYP27B1 significantly decreased with calcitriol treatment (Fig. 4c). The findings confirmed the effect of calcitriol on mRNA expression of vitamin D machinery genes. The expressions of these markers were consistent at least throughout the 2^nd^ to 8^th^ passages of ADMSCs.

**Fig. 5:**
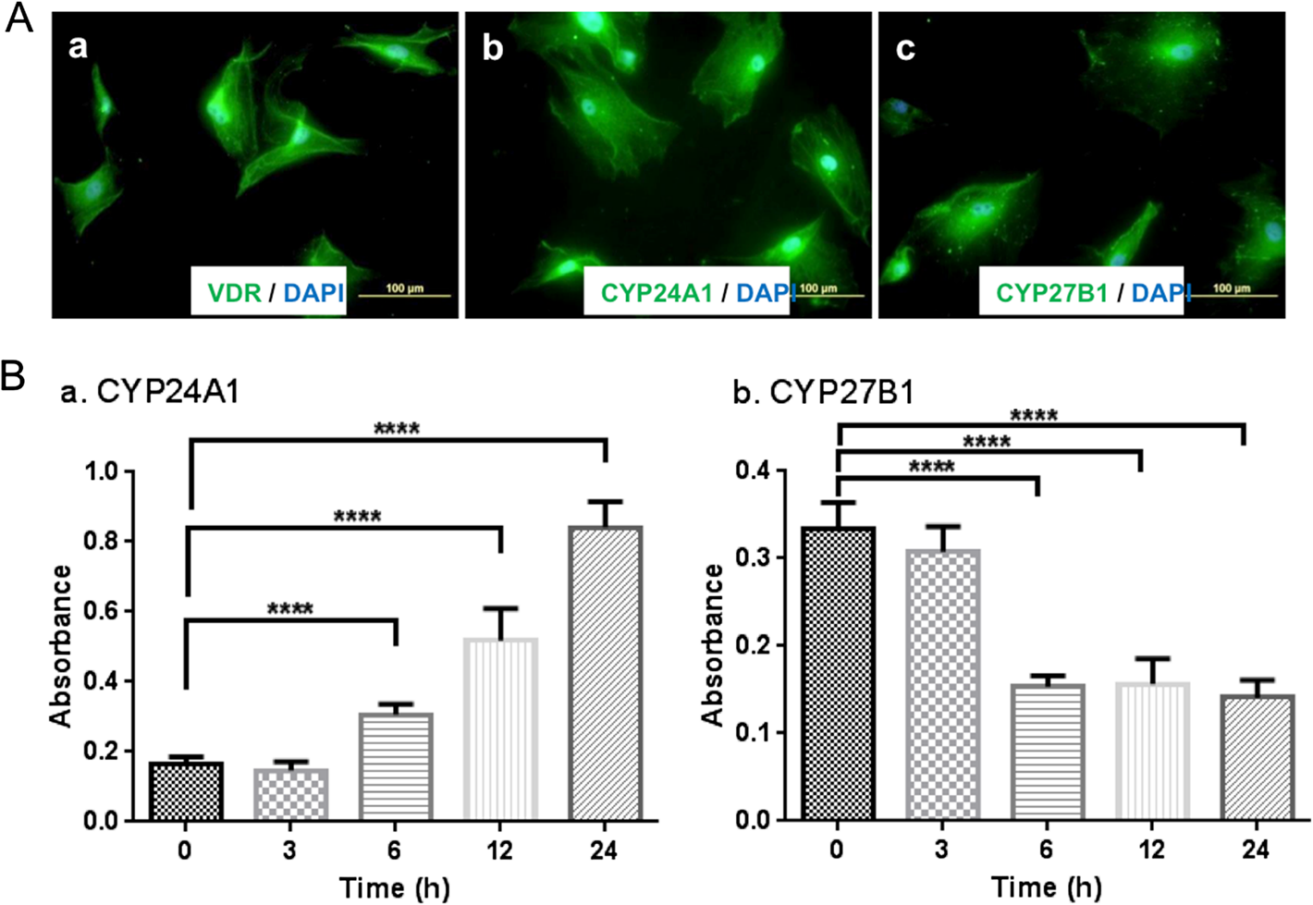
Effect of calcitriol on protein expression of VDR, CYP24A1, and CYP27B1. **a** Phase contrast image showing expressions of VDR (**a**), CYP24A1 (**b**) and CYP27B1 (**c**) after stimulation with calcitriol (10 nM) for 24 hours. **b** ELISA data showing fold change in the expression of CYP24A1 (**a**) and CYP27B1 (**b**) after stimulation with calcitriol (10 nM) for 0 h, 3 h, 6 h, 12 h, and 24 h respectively (^***^ *p* < 0.0001, *n* = 6)

After 24 h stimulation with calcitriol, immunofluorescence demonstrated the presence of VDR, CYP24A1, and CYP27B1 proteins (Fig. 5A). Translation of CYP24A1 and CYP27B1 mRNA into proteins at 0, 3, 6, 12, and 24 h was demonstrated using an ELISA kit (MyBioSource, San Diego, CA, USA). These results correlated with the mRNA data. There was a significant increase in CYP24A1 over 0 to 24 h (Fig. 5Aa, *p* < 0.0001), with the greatest protein expression at 24 h. Furthermore, the expression of CYP27B1 (Fig. 5Bb) correlated with the mRNA data except that the drop in mRNA occurred at 3 h (Fig. 4c), whereas the drop in the protein levels occurred at 6 h (Fig. 5Bb).

**Fig. 6:**
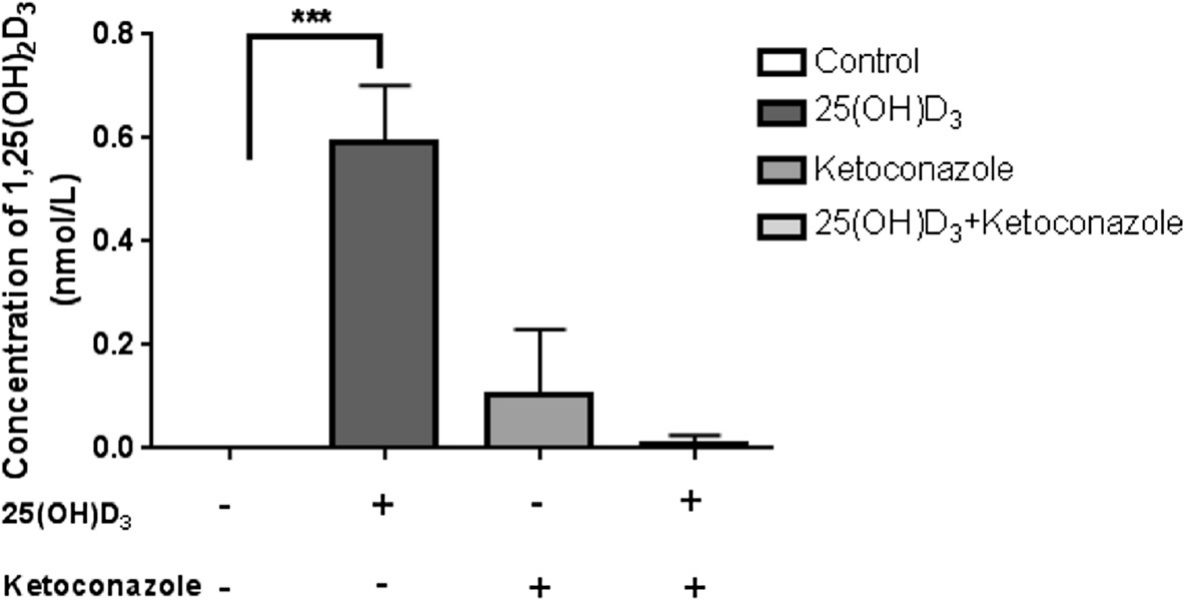
Effect of CYP inhibitor on the conversion of inactive to active vitamin D in ADMSCs. Baseline level of 1, 25(OH)_2_D_3_ in cell culture was measured. ADMSCs were then stimulated with 1 nM 25(OH)D_3_ for 24 hours. Effect of the CYP inhibitor, 1 μM ketoconazole, was examined by itself or in the presence of 1 nM 25(OH)D_3_. (^***^ *p* = 0.001; *n* = 3)

There was no detectable level of 1,25(OH)_2_D_3_ in cell culture without adding 1 nM 25(OH)D_3_ exogenously (Fig. 6). The levels of the active form of vitamin D, 1,25 (OH)_2_D_3_, and CYP27B1 were significantly increased (Fig. 6; *p* = 0.001) in the ADMSCs upon stimulation with inactive form of vitamin D, 25(OH)D_3_. There was mild but insignificant increase in the level of 1,25(OH)_2_D_3_ by ketoconazole (1 μM) in the absence of 25(OH)D_3_. The conversion of inactive vitamin D to active form was almost abolished by 1 μM ketoconazole (Fig. 6).

## Discussion

Mesenchymal stem cells offer a wide range of practical use in regenerative medicine and are currently being investigated for the treatment of diseases, including cardiac [27] and neurodegenerative diseases [28]. ADMSCs are characterized by their adherence to plastic as well as cell surface expression of CD44, CD73, CD90, and CD105, while lacking CD11b+, CD34+, and CD45+. The isolated cells showed strong signals for the group of receptors that are classified as mesenchymal stem cells versus another type of cell that could contaminate the cell culture, such as macrophages, and fibroblasts [29]. However, differentiation into mesoderm derivatives, i.e., osteocytes, chondrocytes, and adipocytes, confirms stem cell classification of the isolated cells [17]. The ADMSCs were also controlled for age-related problems, such as DNA methylation and osteogenic differentiation, since it has been reported that potentially age may alter the ADMSCs [30, 31], by collecting ADMSCs from pigs 5½–6½ months of age, although we found no significant difference in the phenotype of ADMSCs at least up to 24 months age of the pigs. None-the-less, since we used the adipose tissues from pigs of the same age we do not expect much variance. Furthermore, others have reported that ADMSC differentiation remains consistent with age compared to bone marrow MSCs [32]. The isolation process of the ADMSCs from the adipose tissue was thus standardized and established in our laboratory for further experiments on the function and differentiation of ADMSCs.

Strategies for differentiating ADMSCs into different lineages have become a focus due to the therapeutic possibilities. However, not much is known about the effects of micronutrients, including vitamin D, on ADMSCs. The importance of isolating ADMSCs from adipose tissue is stressed due to their high density when compared to the availability of ADMSCs isolated from bone marrow. Adipose tissue is abundant in most individuals and can be harvested using a simple liposuction procedure. This is less invasive and causes less discomfort and damage to the site of trauma. Adipose tissue has a significantly higher stem cell density than in the bone marrow, about a fivefold increase, suggesting that a small amount of adipose tissue can yield sufficient stem cells with proliferation and differentiation potential for autologous cell transplantation, especially in acute clinical settings [18].

Vitamin D deficiency results in low bone density, and in severe cases, osteomalacia. It is also associated with osteopenia, osteoporosis, muscle weakness, and increased probability of bone fracture [33–35]. Vitamin D deficiency has also been reported to be associated with non-skeletal muscle illnesses, such as cancer, autoimmune diseases, infectious disease, and cardiovascular disease [36]. There are two known major sources of vitamin D. One is obtained from dietary intake. The other is chemically synthesized in the skin from 7-dehydrocholesterol. Vitamin D that is obtained through either process goes through multiple steps of action to form 1,25-dihydroxyvitamin D [1,25(OH)_2_D_3_] [37]. This active form is taken up by target cells with the vitamin D receptor (VDR). Vitamin D is primarily activated by CYP27B1 found in the kidney tubule cells. However, additional human cells have been shown to produce [1,25(OH)_2_D_3_], such as vascular smooth muscle cells, endothelial cells, and cardiomyocytes. Finding the VDR and vitamin D hydroxylase in many other tissues suggest that vitamin D hormone acts in an autocrine, paracrine, or an intracrine way to affect the biology of non-classical target tissues [38]. We have demonstrated the presence of the vitamin D machinery; however, the purpose of the machinery in ADMSCs is still not known.

In this study, ADMSC were treated with vitamin D levels similar to those found in the human body. Higher concentrations of 1,25(OH)_2_D_3_ (100 nM) induced cytotoxicity and necrosis. Thus, a 10 nM dose was selected for this experiment due to its low cytotoxicity and its proximity to normal serum levels [39]. During treatment, VDR, CYP24A1, and CYP27B1 are upregulated. The observed increase in the expression of VDR, CYP24A1, and CYP27B1 in response to calcitriol treatment is consistent with the findings in the literature in other cells related to vitamin D metabolism [40–42]. However, the most intriguing and novel finding is that the stimulation of ADMSCs with calcitriol significantly decreased the CYP27B1 expression, while simultaneously increasing CYP24A1 levels. Thus, treatment of ADMSCs with calcitriol inhibits active vitamin D synthesis while also promoting inactivation. This supports our hypothesis that adipose-derived mesenchymal stem cells possess the machinery to metabolize vitamin D. Also, the conversion of cholecalciferol into calcitriol by ADMSCs is a novel finding. This axis promotes cell differentiation or preserves stem cell properties. Further investigation into the function of vitamin D regulation in ADMSCs is warranted and could lead to better understanding of the differentiation process of ADMSCs.

## Conclusions

This is the first report to demonstrate that porcine adipose-derived mesenchymal stem cells contain vitamin D hydrolases and VDR and are able to convert inactive vitamin D into active vitamin D and respond to different concentrations of vitamin D. Hence, in vivo circulating 25-hydroxy vitamin D may have a role in regulating the differentiation of ADMSCs. Further elucidation of ADMSC differentiation has wide clinical potential. Considering their high density and easy isolation, ADMSCs have implications in autologous transplantation in clinical settings.

## Abbreviations

1,25(OH)_2_D_3_: calcitriol
25(OH)D: calcidiol
ADMSC: Adipose-derived mesenchymal stem cells
BSA: Bovine serum albumin
CYP24A1: 24-hydroxylase
CYP27B1: 1-α-hydroxylase
DMEM: Dulbecco’s modified eagle’s medium
FBS: Fetal bovine serum
GAPDH: Glyceraldehyde-3-phosphate dehydrogenase
MSC: mesenchymal stem cell
PBS: Phosphate-buffered saline
VDR: Vitamin D receptor
